# Strategies in Haploidentical Stem Cell Transplantation in Adults

**DOI:** 10.4274/Tjh.2013.0054

**Published:** 2013-12-05

**Authors:** Ulaş D. Bayraktar, Stefan O. Ciurea

**Affiliations:** 1 Department of Stem Cell Transplantation and Cellular Therapy, The University of Texas MD Anderson Cancer Center, Houston, TX, USA; 2 Mercy Cancer Center, Medical Oncology, Hematology, Ardmore, OK, USA

**Keywords:** Haploidentical stem cell transplantation, HLA, GVHD

## Abstract

Haploidentical related donors are alternative stem cell sources for patients without human leukocyte antigen (HLA)-matched related or unrelated donors. Immediate access to the donor, availability for patients with rare haplotypes, ease of stem cell procurement, and lack of a requirement for a physical cord blood bank or an extensive HLA database render this type of hematopoietic stem cell transplantation particularly attractive despite the high histoincompatibility barrier between the recipient and the haploidentical graft. In this review, we answer the following questions: 1) What are the current transplant strategies used to overcome the histoincompatibility barrier in haploidentical stem cell transplantation and their clinical results? 2) How should we choose the donor when there is more than one available haploidentical donor? 3) How does transplantation from haploidentical donors compare to that from umbilical cord blood?

**Conflict of interest:**None declared.

## Abstract (tr)

Tam “human leukocyte antigen” (HLA) uyumlu bağışçı bulunamayan hastalar için bir diğer seçenek yarı-eşlenik akraba bağışçılardan alınacak kök hücrelerdir. Bağışçı ve hasta arasında aşılması gereken yüksek HLA uyumsuzluğuna ragmen, yarı-eşlenik akraba bağışçılarından kan kök hücre nakli (yarı-eşlenik kan kök hücre nakli [YKHN]); bağışçıya anında ulaşılabilirlik, ender gorülen haplotipler icin uygulanabilirlik, kök hücrelerin elde edilmesindeki kolaylık ve kord kanı bankası/doku bankasından bağımsızlığı dolayısıyla cazip bir yontemdir. Bu derlemede şu soruları cevaplandıracağız: 1) YKHN’de HLA uyumsuzluk bariyerini aşmak için kullanılan stratejiler ve sonuçları nelerdir? 2) Birden fazla yarı-eşlenik akraba bağışçısının olması durumunda bağışçı nasıl seçilmelidir? 3) YKHN’in korddan kök hücre nakline göre avantaj ve dezavantajları nelerdir?

## INTRODUCTION

Two-thirds of patients who require allogeneic hematopoietic stem cell transplantation (SCT) do not have a human leukocyte antigen (HLA)-matched related donor available [1]. A matched unrelated donor can be identified in only 50% to 60% of these cases. The chance of finding such a donor is particularly poor for patients whose ethnicity is under-represented in HLA databases. Haploidentical donors – parents, children, and half of siblings – are alternative stem cell sources for such patients without matched donors. The first successful SCT from a haploidentical donor (haploSCT) was reported in 1981 in a 10-month-old infant using an ex vivo T cell-depleted bone marrow graft from her father [2]. After 30 years of experience, transplanters are now better at overcoming the histoincompatibility barrier between the recipient and the haploidentical donor. 

**What are the current transplant strategies used to overcome the histoincompatibility barrier in haploSCT and their clinical results?**

For successful haploSCT, both the patient’s and the graft’s immunity should be suppressed or modified to prevent graft failure and graft-versus-host disease (GVHD). Various strategies have been devised to achieve the required suppression without substantially increasing treatment-related mortality (TRM) arising from immunosuppression. These strategies may be studied in 2 groups: those utilizing ex vivo T cell-depleted grafts and those utilizing T cell-replete grafts. 

With currently available magnetic selection methods, 3 to 5 logs of ex vivo T cell depletion (TCD) of the stem cell graft is possible [3], and this is the most effective method to prevent GVHD after SCT. Unfortunately, extensive TCD of the graft impairs engraftment and increases primary graft failure rates as more host immune cells survive post-SCT. In initial trials, T cell-depleted grafts from haploidentical donors were rejected in up to 50% of cases [4]. The risk of graft rejection may be reduced by intensification of the conditioning regimen [5,6], in vivo host TCD with antibodies [7], and increasing of the bone marrow (BM) inoculum (number of CD34+ cells infused) [8]. The most notable haploSCT protocol to date was devised at the University of Perugia in the 1990s, in which a “mega-dose” of CD34+ cells (while a threshold for the dose has not been defined, the reported minimum is 5.1x106 CD34+ cells/kg) derived from BM and peripheral blood after TCD was used with ablative conditioning and anti-thymocyte globulin [3,9]. While GVHD incidence was minimal and the graft rejection rate was acceptable, TRM due to infections remained an issue, which is the current focus of transplanters utilizing TCD grafts. Although ex vivo TCD in haploSCT is most commonly achieved by positive selection of CD34+ cells, negative selection of lymphocyte subsets through CD3/CD19 or TCRαβ retains other donor immune cells, i.e. natural killer (NK) cells, that may decrease the incidence of GVHD and exert a graft-versus-leukemia effect [10]. The strategies used in TCD haploSCT are summarized in Table 1 (Table 1 Contunied) with their respective clinical results. 

Without TCD of the graft, a higher-intensity GVHD prophylaxis regimen or selective inhibition of graft T cells becomes necessary to prevent GVHD after haploSCT. While Chinese researchers chose to intensify immunosuppression and prime the BM graft with granulocyte colony-stimulating factor (G-CSF) [11], researchers from Johns Hopkins led the way by selectively inhibiting graft immunity against donor cells using post-SCT cyclophosphamide [12,13]. One of the more established methods to be utilized in haploSCT, which was studied and reported in a recent Blood and Marrow Transplant Clinical Trials Network (BMT CTN) trial [14], post-SCT cyclophosphamide has little impact on stem cells and engraftment while primarily targeting donor lymphocytes activated by recipient antigens immediately after graft infusion. The rationale and clinical results of haploSCT strategies not utilizing TCD of the graft are summarized in Table 1(Table 1 Contunied). 

Overall, while TCD results in lower GVHD incidence with acceptable engraftment rates when a “mega-dose” of CD34+ cells is used, a relatively high TRM rate primarily due to infections remains an issue. Furthermore, TCD requires an initial investment in facilities employing good manufacturing practice with cell selection instruments, i.e. CliniMACS, and expertise to run such facilities. The initial investment cost may be difficult to attain in developing and under-developed countries where haploSCT would be particularly valuable since residents of such countries are generally under-represented in international HLA databases. While haploSCT with T cell-replete grafts may lead to higher GVHD incidence, it allows the intensity of conditioning regimens to be reduced through host immunity suppression utilizing engraftment. However, the reduced intensity conditioning regimen used in most studies of post-SCT cyclophosphamide may lead to high relapse incidence in acute leukemic patients. At the MD Anderson Cancer Center, we compared the outcomes of haploSCT with TCD peripheral blood grafts to that with unmanipulated BM grafts after an identical ablative conditioning regimen (fludarabine-melphalan-thiotepa) [15]. Early results revealed significantly higher rates of overall and progression-free survival with unmanipulated BM grafts, primarily because of significantly lower TRM (16% vs. 42% at 1 year). 

**How should we choose the donor when there is more than one available haploidentical donor?**

Most patients requiring SCT have more than one haploidentical donor. The presence of recipient antibodies against donor-specific HLA, killer immunoglobulin-like receptor (KIR) mismatch predicting NK cell alloreactivity, mismatch for non-inherited maternal vs. paternal alleles, degree of HLA mismatch between donor and recipient, vdonor age, and ABO-match should be taken into account while deciding on the donor among available haploidentical candidates. 

Transplant recipients may have developed anti-HLA antibodies against donor HLA antigens (donor-specific antibodies; DSAs) during pregnancy or after blood product transfusions. The presence of DSAs is associated with increased risk of primary graft failure after SCT [16,17,18,19]. Additionally, the level of DSAs in recipient serum is likely important. If a patient has DSAs against all haploidentical donors, selecting donors with the lowest antibody level may be appropriate. Treatment of recipients with plasma exchange or rituximab may also be reasonable and has been used in solid organ transplantations. 

NK cells primarily attack hematopoietic cells, sparing solid organs [20]. In recipients lacking HLA class I alleles specific to the donor KIRs, donor NK cells may prevent GVHD and disease relapse by eliminating residual recipient antigen-presenting cells and leukemia cells [21,22]. Accordingly, KIR mismatch between recipient and donor has been associated with improved haploSCT outcomes [21,22,23]; however, this finding has been disputed by other researchers [24,25]. KIR mismatch may play a more pronounced role in SCT for myeloid malignancies [22,26]. Further studies are needed to verify the impact of NK alloreactivity and KIR mismatch on haploSCT outcomes. 

Although a progressive increase in TRM with increasing genetic disparity has been historically reported, contemporary transplant strategies may negate this correlation by overcoming larger histoincompatibility barriers. In fact, Kasamon et al. reported no increased incidence of acute GVHD (aGVHD) and non-relapse mortality (NRM) after haploSCT from full-haplotype mismatched donors compared to those with better-matched donors [27]. Moreover, patients with more than 3 mismatches appeared to have better outcomes due to a lower relapse incidence. 

Immunologic tolerance may develop between mother and fetus during pregnancy [28,29], leading to down-regulated immune responses if the mismatched haplotype between the recipient and the haploidentical donor is of maternal origin. Accordingly, patients with maternal donors were found to survive longer than those with paternal donors [30], and TRM was reported to be lower in patients with recipients mismatched for non-inherited maternal HLA compared to those with recipients mismatched for paternal antigens [31]. 

The immune system is subject to senescence with advancing age. Although no data exist on an association between donor age and outcomes after haploSCT, the findings of higher GVHD incidence and shorter survival after unrelated donor transplants from older donors compared to younger donors would probably apply for haploSCT, as well. Older multiparous women may be the least preferred donors for male recipients [32].

Studies have demonstrated that infusion of larger numbers of CD34+ cells improved outcomes after SCT [33,34,35]. Stem cell dose is also likely important in haploSCT, as can be inferred from the improved outcomes with mega-doses of peripheral blood stem cells in TCD haploSCT [9]. Transplants involving a major ABO incompatibility require mononuclear cell separation to prevent a hemolytic reaction, which reduces the graft cell dose. If maximizing the infused stem cell dose is indeed important in haploSCT, then younger, larger donors without a major ABO incompatibility with the recipient should be preferred. 

**Figure 1:**
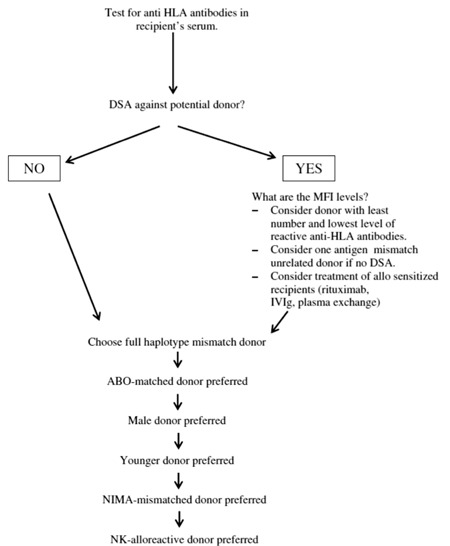
Proposed algorithm for donor selection in haploidentical stem cell transplantation. DSA indicates donor-specific anti-HLA antibodies; MFI: median fluorescence intensity, NIMA: non-inherited maternal antigens, NK: natural killer. Reproduced from Ciurea and Champlin with permission (32).

An in-depth review of donor selection in haploSCT is available from Ciurea and Champlin [32] and the proposed algorithm is shown in Figure 1. 

**How do transplants from haploidentical donors compare to those from umbilical cords?**

For patients lacking an HLA-matched related or unrelated donor, umbilical cord blood (UCB) is another alternative stem cell source. UCB is more immune-plastic than peripheral blood and bone marrow grafts; therefore, 2 or 3 out of 6 HLA mismatches are allowed for UCB transplants. However, use of UCB as a stem cell source has been limited until recently by the delayed engraftment and relatively high rate of primary graft failures due to the low volume and low CD34+ cell content. Use of double, instead of single, UCB has partially overcome these issues [36,37].

The advantages and disadvantages of haploSCT and UCB SCT are outlined in Table 2. Although they had not been systematically compared to each other, a recent parallel multi-center phase 2 trial by BMT CTN confirmed the utility of both double UCB and haploidentical donors as alternative stem cell sources [14]. Fifty patients in each arm, with advanced hematological malignancies, received either BM grafts from haploidentical donors or double UCB after similar conditioning regimens including fludarabine, cyclophosphamide, and low-dose total body irradiation (TBI). Grade II-IV acute GVHD and chronic GVHD incidences were numerically higher in the double UCB arm (40% vs. 32% and 25% vs. 13%), demonstrating efficacy of the post-SCT cyclophosphamide in the haploSCT arm. NRM at 1 year was 24% and 7% in the double UCB and haploSCT arms, while relapse incidence was 31% and 45%, respectively. One-year progression-free survival (PFS) was similar in both arms at 46% and 48%. Similarly, a retrospective analysis of the European Group for Blood and Marrow Transplantation (EBMT) database demonstrated significantly lower acute GVHD rates after haploSCT compared to UCB SCT between 1998 and 2002 [38]. A randomized BMT CTN study is ongoing in the United States, comparing SCT from haploidentical donors and UCB in patients with hematological malignancies. 

With our current knowledge, it is difficult to recommend one stem cell source over another for patients without matched donors. Until a large-scale randomized prospective study shows one’s superiority, transplant centers will and should choose an alternative stem cell source based on their own expertise. However, T cell-replete haploSCT is clearly advantageous for countries and centers without the financial backing to invest in and maintain an umbilical cord bank. Despite these advantages and recent advances, haploSCT is a risky procedure with additional perils of late-onset chronic GVHD and infections due to the histoincompatibility barrier, late immune reconstitution, and intensified GVHD prophylaxis limiting its use to experienced centers. **Conflict of Interest Statement** The authors of this paper have no conflicts of interest, including specific financial interests, relationships, and/ or affiliations relevant to the subject matter or materials included. 

## Figures and Tables

**Table 1:**
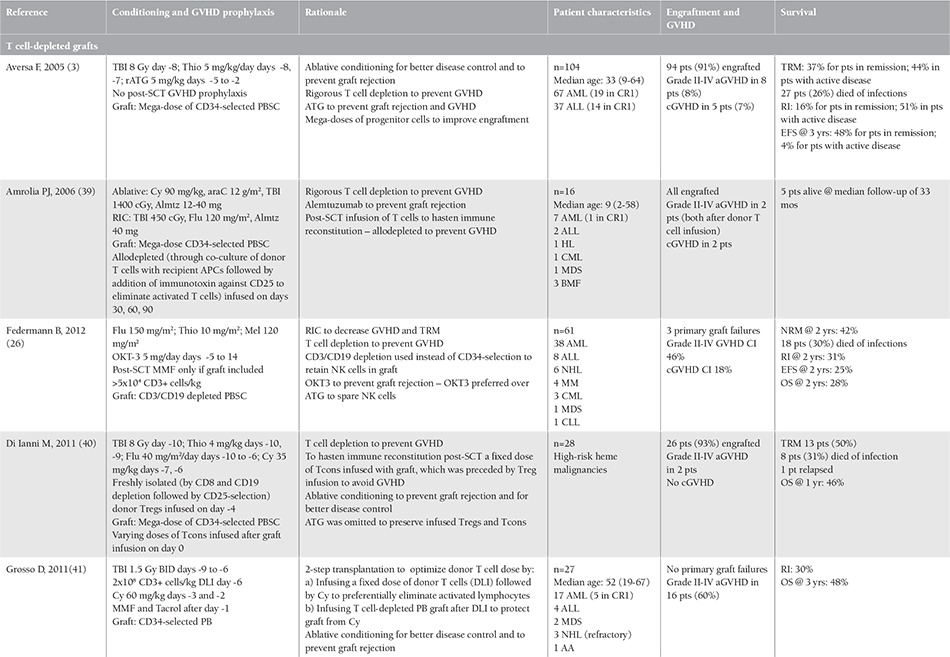
Studies utilizing different strategies to overcome histoincompatibility barrier in hematopoietic stem cell transplantation from haploidentical donors.

**Table 1 Contunied:**
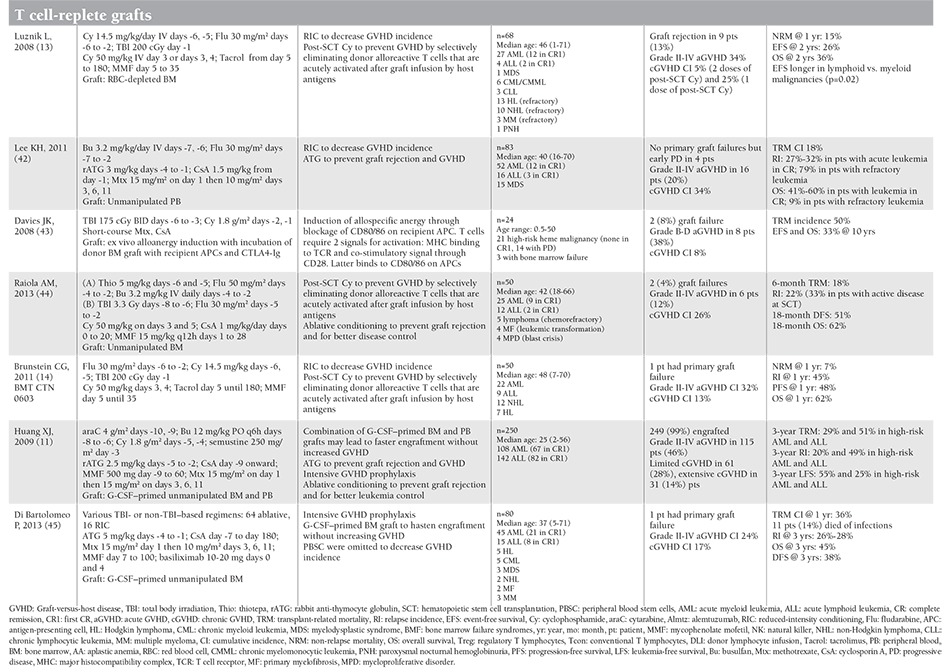
Studies utilizing different strategies to overcome histoincompatibility barrier in hematopoietic stem cell transplantation from haploidentical donors.

**Table 2:**
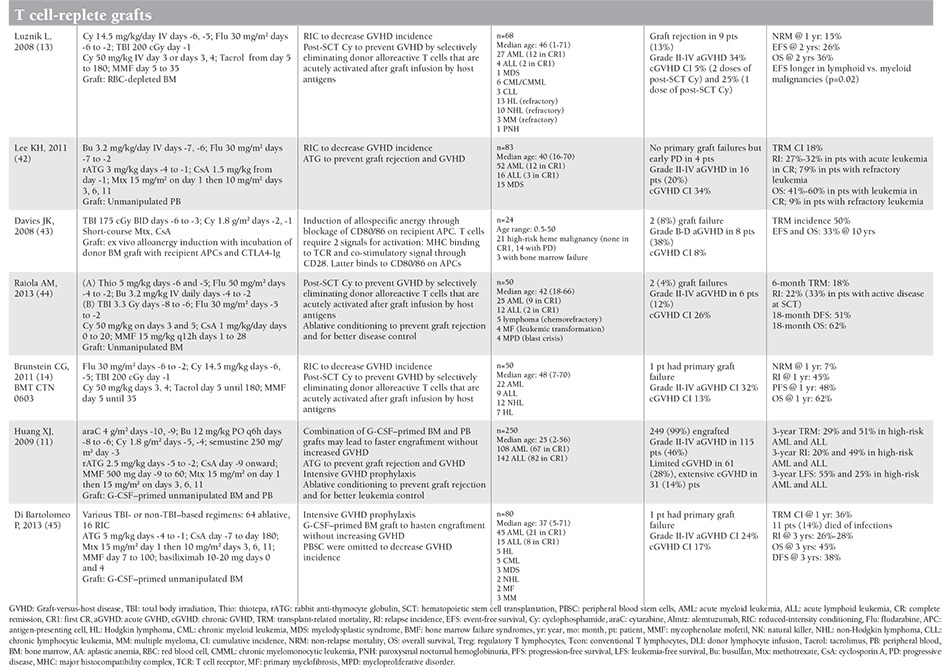
Comparison of hematopoietic stem cell transplantation from umbilical cord and haploidentical donors
